# TCR-like CARs and TCR-CARs targeting neoepitopes: an emerging potential

**DOI:** 10.1038/s41417-021-00307-7

**Published:** 2021-03-02

**Authors:** Mansour Poorebrahim, Niloufar Mohammadkhani, Reza Mahmoudi, Monireh Gholizadeh, Elham Fakhr, Angel Cid-Arregui

**Affiliations:** 1 Targeted Tumor Vaccines Group, Clinical Cooperation Unit Applied Tumor Immunity, German Cancer Research Center (DKFZ), Heidelberg, Germany; 2 Department of Clinical Biochemistry, School of Medicine, Shahid Beheshti University of Medical Sciences, Tehran, Iran; 3 Cancer Immunology Project (CIP), Universal Scientific Education and Research Network (USERN), Tehran, Iran; 4 Department of Medical Biotechnology, Faculty of Advanced Medical Sciences, Tabriz University of Medical Sciences, Tabriz, Iran; 5 Department of Immunology, Pasteur Institute of Iran, Tehran, Iran; 6 Faculty of Biosciences, Heidelberg University, Heidelberg, Germany

**Keywords:** Cancer immunotherapy, Translational immunology

## Abstract

Neoepitopes or neoantigens are a spectrum of unique mutations presented in a particular patient’s tumor. Neoepitope-based adoptive therapies have the potential of tumor eradication without undue damaging effect on normal tissues. In this context, methods based on the T cell receptor (TCR) engineering or chimeric antigen receptors (CARs) have shown great promise. This review focuses on the TCR-like CARs and TCR-CARs directed against tumor-derived epitopes, with a concerted view on neoepitopes. We also address the current limitations of the field to know how to harness the full benefits of this approach and thereby design a sustained and specific antitumor therapy.

## Introduction

Malignancies result from the accumulation of a variety of mutations and epigenetic changes. Mutations can be appeared in cell surface proteins or presented as neoepitopes by the major histocompatibility complex (MHC), making tumor cells detectable by immune cells [1]. Nonetheless, tumor cells usually escape from the immune cells and thereby gain the opportunity to develop and invade. Major mechanisms involved in immune evasion include reduced immune recognition through the loss of tumor antigens and expression of cytokines (e.g., VEGF, IL-10, TGFβ) or immunoregulatory molecules (e.g., IDO and B7 family checkpoint molecules), that lead to the induction of an immunosuppressive tumor environment and enhance tumor resistance or survival via elevated expression of STAT3 or of BCL-2 [2, 3].

Engineered T cells with selective antigen receptors are highly potent to identify and destroy tumor cells in efficient manner [3]. In this context, the main approaches include chimeric antigen receptors (CARs), T cell receptor (TCR) engineered T cells (TCR-Ts), TCR-like CARs, and TCR-CARs. CARs are chimeric molecules engineered to recognize a tumor antigen, leading to the MHC-independent activation of CAR T cell. Since most of the tumor mutations, including neoepitopes, are presented on the cell surface through MHC class I molecules, there is a limitation of target selection for CAR design. This limitation can be overcome by using approaches based on the TCRs such as TCR-like CARs, TCR-CARs [4, 5]. Unlike CARs, TCRs are not restricted to the cell surface antigens, but can detect and bind to the peptides presented by MHC molecules (pMHCs). This feature provides a wide range of potential targets for TCRs such as tumor-specific neoepitopes. Of note, redirection of TCR-based CARs on the highly tumor-specific neoepitopes can prevent “off-tumor” toxicities that are commonly associated with CAR therapies [3]. However, rearrangement of the native and transduced TCR’s αβ chains may cause autoimmune reactions because of their unknown specificity [6]. Thus, the combination of TCR and CAR features will apparently result in more benefits. TCR-like CARs comprise an extracellular single-chain variable fragment (scFv) arising from TCR-like antibodies which recognize tumor-associated pMHCs in the same way as full TCRs do [7]. This approach does not suffer from the problem of competition and rearrangement with endogenous TCRs, and possess many potential pMHC targets. In TCR-CARs, the variable domains of TCR (TCRv) recognizing pMHC are linked to intracellular domains of CARs [4].

## Neoepitopes

Over the recent years, tumor-specific neoepitopes lay the path forward for the personalized immunotherapy approaches. Almost all cancers display various genetic alterations (such as single-nucleotide variants (SNVs), insertions and deletions (indel), gene fusions, frameshift mutations, and structural variants (SVs)) [8], a minority of which may result in somatic tumor mutations harboring elegant tumor specificity. These “neoepitopes” are foreign in nature and presented on MHC class I or II molecules, and in case they can provoke immune reactivities through putative mechanisms, are referred to as “neoantigens” [9, 10]. Here, we actually refer to the antigenic neoepitopes when discussing neoepitopes. Identification of different neoepitope repertoires in cancer patients who have reaped the therapeutic benefit compared to the patients with immunotherapy resistance consequences could provide beacons for an efficient choice of ideal neoepitopes for therapy. Of note, neoepitopes’ qualitative characteristics, which may result in a vigorous and sustained immunity, is of remarkable importance. In the first instance, untranslated mutations could obviously not evoke an immune response, therefore, any signs of transcript downregulation including, promoter methylation, exon skipping as well as chromatin remodeling should take into account during the characterization of prospective mutations as they might open the opportunity of immune evasion [11]. HLA loss is another well-reported mechanism of immune escape and subsequent resistance during tumor progression. Thus, it would be of high value to classify multiple HLA-binding neoepitopes as a high priority since it may prevent the resistance evolvement to neoepitope-targeted therapies due to the HLA loss [12]. The clonal fraction is the other key feature of neoepitopes that should be considered. Evidence supports the fact that effective responses to checkpoint inhibitor therapy are correlated with the burden and fraction of clonal neoepitopes [13]. By their nature, clonal neoepitopes are expressed by a higher cancerous cell fraction compared to sub-clonal neoepitopes. Moreover, every cancer cell does not express sub-clonal neoepitopes, hence, they potentially possess less chance for efficient immune control within all parts of the disease [12]. Not to mention that similarity to the self or known antigens is also worth to be noted. Due to the fact that the immune system is equipped with the capability to recognize non-native antigens, “non-self” degree of a peptide can affect the probability of efficient immune control [12]. Importantly, the vast majority of potential mutations are typically accounted for passenger rather than driver events and their loss via chromosomal instability during tumor growth could be commonly tolerated. However, mutations in cancer driver and cell survival-associated genes are considered as essential neoepitopes. According to the available evidence, multiple HLA-binding clonal neoepitopes expressing in crucial genes that could not be deleted or repressed owing to their position in the genome might be considered as the high-quality neoepitopes. Such targets could apparently serve as potential means of immune surveillance and immunotherapy approaches such as adoptive T cell therapy.

**Fig. 1:**
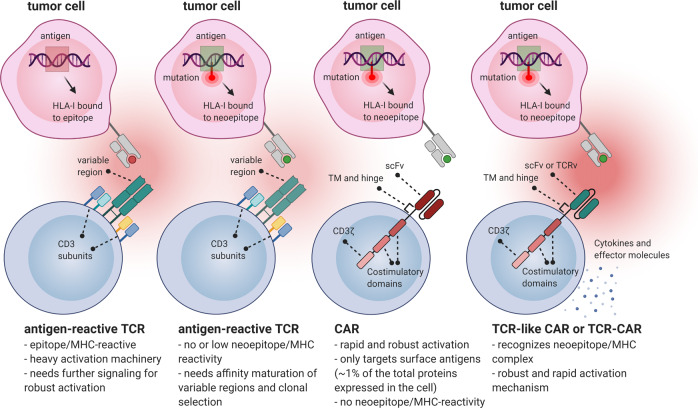
Neoepitope generation and targeting. Conventional TCRs can recognize cognate pMHC complexes, but there is a need for further co-stimulatory signaling to fully activate T cells. When a mutation occurs in the tumor antigen, the previous tumor epitope-reacting TCRs have no/low affinity to the generated neoepitope. In order to recognize the neoepitope/MHC complex, the variable region of TCR undergoes affinity maturation process, and further TCR clonal selection is occurred. Neoepitope-reactive TCR variable domains (TCRv) can be further used in CAR structure. Although CAR T cells (here a 3rd generation CAR) are rapidly and strongly activated when stimulated by the target antigen, they can only target cell membrane surface proteins which are ~1% of total proteins expressed in the cell. Therefore, CARs are unable to target neoepitopes in the context of major histocompatibility complex (MHC) molecules. However, TCR-like CARs (with a scFv targeting pMHC) or TCR-CARs (with a TCRv targeting pMHC) can be developed to have the advantages of both TCRs (for targeting neoepitopes) and CARs (for rapid and robust activation).

To distinguish neoepitopes, TCRv undergoes affinity maturation and subsequent selections. Therefore, in addition to neoepitopes, it is also critical to characterize neoepitope-reactive TCRv that can be used in the TCR-based therapies (Fig. 1). Neoepitope identification is technically complicated and affected by the current approaches. Using tumor and normal DNA, whole-exome sequencing (WES) is recruited to characterize tumor-specific non-synonymous mutations (NSM). When possible, RNA-seq is also employed to choose expressed mutations [14]. After the identification of NSMs, some strategies are exploited to select the list of candidate neoepitopes that will be subsequently evaluated for immunogenicity. In the WES data-based strategy, unfiltered candidate neoepitopes are listed and identified. However, there is a high success rate for tumors with a high mutation load [15]. Mass spectrometry (MS)-based immunopeptidomics is another technique which not only provides the characterization of post-translational modified peptides and non-canonical neoepitopes but also directly identifies naturally presented HLA-bound peptides [16, 17]. Nonetheless, the MS-based method is less sensitive and depends on the expression of HLA in cancerous cells. Besides, it requires a large number of samples from tumor tissue. In silico peptide prediction is also used to increase the reliability of candidate neoepitopes. However, it is not ideal for HLA class II-presented peptides, and also some peptides might be imprecisely predicted [18]. Although prompt and accurate identification of authentic neoepitopes in any given patient remains an obstacle, the technological advances and innovative screening assays might be promising for effective translation of neoepitopes targeting into more beneficial treatment strategies for cancer patients [18].

## Adoptive therapies targeting neoepitopes

Recent technological advances in genome sequencing and T cell engineering have paved the way for the development of adoptive cell therapy (ACT) targeting multiple cancer-specific mutations. Avoiding vital healthy tissue damage, neoepitope-reactive T cell administration has revealed clinical benefits for patients suffering from advanced solid tumors [19].

In this strategy, tumor specimens are used to provide tumor-infiltrating lymphocytes (TILs) containing CD8 and CD4 T cells, applying to recognize tumor-associated antigens and TCR sequences. The designed experiment of Stevanovic et al., indicated that the application of tumor-infiltrating adoptive T cell therapy in patients with papillomavirus-associated metastatic cervical cancer led to the entire cancer regression. Interestingly, mutated neoepitopes or a cancer germline antigen (KK-LC-1) caused the prominent T cell reactivities compared to the common viral antigens, suggesting a novel landscape of directing nonviral targets in immunotherapy of virally mediated cancers. Importantly, PD-1 expression was significantly observed in both viral and nonviral tumor antigen-specific T cells, indicating that antitumor T cell reactivities might be unleashed by PD-1 blockade strategies [20]. In a study of chemo-refractory metastatic breast cancer, TILs were directed against neoepitopes formed by non-synonymous mutation of four proteins: SLC3A2, KIAA0368, CADPS2, and CTSB. Administration of the neoepitope-specific TILs in combination with IL-2 and a brief course of checkpoint blockade (pembrolizumab) substantially provoked mutation-specific polyclonal responses and consequently led to the complete regression of cancer for more than 22 months [21]. Efforts are more recently underway to employ oncogenes and tumor suppressor gene mutations, so-called “hotspot” mutations, for validation of potential neoepitopes. For instance, objective tumor regression in a patient with metastatic colorectal cancer was recorded following polyclonal CD8 T cell reactivities against mutant-type K-Ras (K-Ras^G12D^). However, the lack of expression of the HLA-C*08:02 molecule in a single lesion mediated tumor immune escape and resulted in tumor progression nine months after the treatment [22]. Furthermore, in addition to characterization of T cell responses targeting mutant-type K-Ras variants (G12D and G12V), Cafri and colleagues [23] identified CD8 memory T cells recognizing mutant-type SMAD5 and MUC4 proteins in the peripheral blood of patients with metastatic colon cancer, which could be potentially recruited to develop efficient personalized cancer immunotherapy based on neoepitope-reactive T cells. The most common mutated gene in cancer, TP53, is also an ideal candidate for assessment of targeted cancer immunotherapy due to its immunogenic potential. The most common hotspot mutations in TP53 (eight positions) were detected in 24% of common epithelial cancers (133 patients). T cell reactivities against the p53 neoepitopes presented through both intracellular and extracellular pathways were systematically and thoroughly analyzed using autologous antigen-presenting cells (APCs) expressing entire HLA class I and II molecules. This study laid the foundations for ongoing clinical trials evaluating the capability of TP53 mutation-specific TILs and TCRs to eradicate metastatic cancers [24].

Despite perceptible developments, targeting neoepitopes through adoptive therapy approaches yet is facing serious challenges regarding tumor cells and their microenvironment. Heterogeneity is a critical issue, as antigen processing and target presentation by tumor cells are variable. In other words, it must be taken into special consideration that not every “neoepitope” creates “neoantigen” that can be practically distinguished and trigger efficient T cell reactivities when presented by APCs with sufficient MHC-peptide expression [25]. In addition, infiltration and trafficking of cytotoxic T cells (CTL) into tumor is essential for their function and there are different approaches to enhance the homing of genetically modified T cells into the tumor microenvironment. For instance, the level of CXCL8/IL-8 is increased in the melanoma tumor microenvironment, and engineered MAGR-A3 TCR-T cells expressing CXCR2 have shown higher infiltration in xenograft murine models [26]. Immune checkpoint inhibitors can also increase CAR T cells’ efficacy and hinder the immune-suppressive tumor niche [27]. Besides that, the suppressive extracellular matrix (ECM) of tumors can be overcome by the expression of degrading enzymes such as Heparanase in CAR T cells [28], which can be applicable for TCR-CAR-based adoptive therapies.

## TCR-like CAR and TCR-CAR

**Fig. 2:**
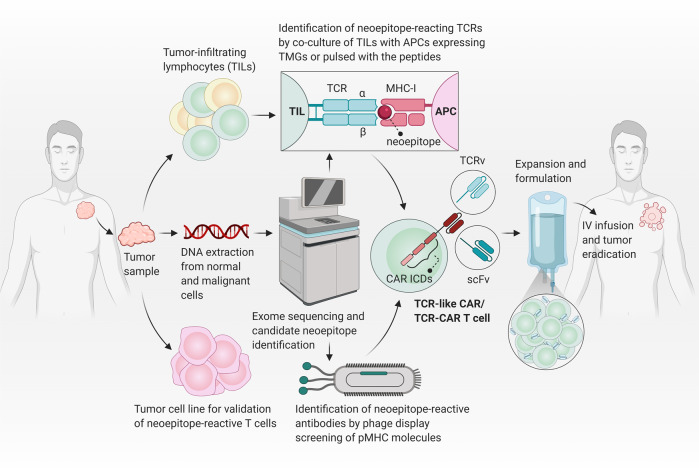
Current methodologies to identify neoepitopes and develop TCR-like CARs or TCR-CARs. First, a tumor is surgically excised from the patient and tumor samples are used as the subject for performing whole-exome sequencing and identifying tumor-specific non-synonymous mutations that occurred in coding regions. In parallel, tumor-infiltrating lymphocytes (TILs) and tumor cell lines are also obtained from tumor samples to identify and validate neoepitope-reactive TCRs. The predicated neoepitope sequences surrounding each mutation by flanking regions of 12 codons on either side are synthesized as tandem minigenes (TMGs; constructs encoding multiple neoepitopes each encoding a specific mutation) or single long peptides with 25 amino acid lengths. TILs are co-cultured in the presence of an antigen-presenting cells (APC) expressing the TMG or pulsed with the long peptides. In parallel, the neoepitope/HLA monomers can be sued for isolation of TCR-like antibodies targeting the cognate neoepitope/HLA complex. Finally, characterized TCR variable domains (TCRv) or single-chain variable fragment (scFv) specific for neoepitope/MHC complex are used in a CAR structure to produce TCR-CARs or TCR-like CARs, respectively. The modified T cells can be further expanded and formulated in a proper buffer and infused to the patient by intravenous (IV) injection to specifically destroy tumor cells. CAR ICDs CAR intracellular domains.

Genetically manipulation of TCRs has provided the basis of CARs development. CARs are synthetic receptors that typically contain CD3ζ as the intracellular domain, responsible for downstream signaling plus a co-stimulatory domain(s) which is commonly CD28 or 4-1BB, and an extracellular antigen-binding domain that is an scFv derived from an antibody interacting with unprocessed antigens including cell surface-expressed proteins, glycolipids, and carbohydrates. However, this recognition is in a MHC-independent manner, which limits CAR T cells’ recognition capability only to cell surface antigens (~1% of the whole cell’s expressed proteins). Nonetheless, TCRs are composed of an αβ transmembrane heterodimer and CD3 subdomains and possess the advantage of MHC-dependency. Thus, they are able to target any peptide derived from cellular protein degradation. In other words, the whole proteome can be recognized by TCRs [29]. Although TCR-T cells with defined specificity have exhibited treatment efficiency, the competition of exogenous therapeutic TCR with the endogenous TCR for CD3 signaling will augment the possibility of formation of mispaired dimers, which may subsequently lead to the unfavorable specificity and function. Importantly, TCR localization is restricted to T cells, as T cells prepare all substituents for effective TCR induction. While other immune cells like natural killers (NKs) and macrophages also can be engineered to express CAR construct [30, 31]. Moreover, compared to CARs, TCRs are contrarily characterized by almost lower affinity owing to the fact that high-affinity TCR-expressing T cells are subjected to negative selection in the thymus, as the majority of tumor antigens are “self” proteins but typically overexpressed. Accordingly, further advances in the area of immunotherapy depend in part on promoting the functional capacity of the engineered components. In this regard, the development of TCR-like CAR T cell is a novel strategy. In this approach, a new mode of epitope/MHC complex-specific antibodies (known as TCR mimic/TCR-like antibodies) are developed to bind MHC-bound targets. TCR-like antibodies were designed with almost 10^3^–10^5^ times stronger affinity compared to the natural TCRs. Furthermore, the CAR construct can convey its own activation signals through embedded transmembrane and intracellular domains without competition with endogenous TCR for signaling domains [5]. This approach primarily was introduced by Willemsen and coworkers in 2001 [32] when a CAR construct linked with a TCR-like antibody against MAGE-A1/HLA-A1 complex. On the other hand, affinity enhancement of TCRv through amino acid replacement can improve their MHC-bound target recognition and interaction. TCRv structure can also be linked to the intracellular signaling domains of CAR construct, generating TCR-CAR [4]. These novel structures are being improved to simultaneously represent favorable characteristics of both TCRs and CARs. Figure 2 shows a schematic illustration of the recent procedures of neoepitope characterization and development of neoepitope-reactive TCR-like antibodies or TCRv applied in TCR-like CAR or TCR-CAR platforms, respectively.

## TCR-based CARs against tumor epitopes/neoepitopes

In contrast to the “self” epitopes derived from shared or overexpressed antigens, neoepitopes can be potentially expressed at far higher rates and be recognized by highly tumor-specific TCRs [33]. Accordingly, TCR-like CARs and TCR-CARs against MHC molecules bound to a tumor antigen-derived epitope/neoepitope have been proven to be very efficient. For instance, Wälchli and colleagues constructed two TCR-CARs against DMF5 (from MELAN-A antigen) and Radium-1 (from a mutated form of TGFβR2). Both TCR-CARs, particularly Radium-1 TCR-CAR, stably expressed and redirected their host cell on cognate pMHC, where the engineered cell could efficiently kill target cells [4].

Historically, the β-chain was the topic of interest for the majority of researchers studying in the TCR biology field. This is apparently due to the higher capacity of β-chain in generating diversity (because of D gene component) as well as its unique expression in each single cell, where the expression of two different α chains can be observed in the same cell [34, 35]. In an interesting approach, the single variable domain of the β-chain (Vβ) targeting NY-ESO-1 and MAGE-A3 antigens were constructed and assessed in the structure of TCR-CAR and TCR. The TCR-CAR Jurkat cells carrying only Vβ domains as extracellular compartment showed a dose-dependent activation in the co-culture of serially diluted peptides and APC. This confirms the feasibility of Vβ-only TCR-CAR constructs as β-chain can ideally mimic whole TCR [36].

Recently, TCR-like antibodies targeting pMHC complexes have shown strong antitumor effects and many of these compounds are now under development in pre-clinical settings [37]. It has been previously established that the application of antibody-derived moieties as the antigen-recognition domain of CARs can substantially eradicate tumor cells with down-regulated antigen expression (~200 copies/cell) [38]. Therefore, these structures can also be used in TCR-like CAR structure. In this context, Sadelain’s group generated TCR-like CAR T cells restricted to HLA-A2/NY-ESO-1_157–165_. However, they found that despite the specificity of high-affinity Fab fragments in soluble form, the TCR-like CAR T cells expressing the Fab extracellular domain showed only moderate lysis of HLA-A2/NY-ESO-1_157–165_ expressing targets. The authors hypothesized that this might be because of the strong binding affinity of the Fab to HLA-A2 molecules. Thus, they lowered the Fab’s HLA-A2 binding affinity to TCR level by a rational mutagenesis approach, and thereby, improved the specificity and efficacy of TCR-like CAR T cells [39]. In another antibody-based TCR-like CAR design, Akahori et al., designed a TCR-like CAR with low affinity (*K*_d_ = 741 nM) against HLA-A*2402/WT1_235–243_ complex. The study confirmed the therapeutic efficacy of this strategy. Importantly, the authors indicated that scFvs with higher binding affinity to WT1_235–243_/A*2402 (*K*_d_ = 34.4 nM) had lower effector function in WT1^+^/A*2402^+^ tumor cell lines (probably with low antigen load). Therefore, they suggested an optimum binding affinity of TCR-like CARs to the pMHC as a crucial criterion for the serial triggering of the target antigen (quick release of TCR-like CAR from the pMHC) and facilitating modified T cell activation [40]. In support of this notion, Oren et al. [41], found that TCR-like CAR T cells containing a high-affinity scFv recognizing HLA-A2-WT1_Db126_ exhibited lower effector functions and loss of specificity compared to the engineered cells expressing low-affinity αβ-TCRs. However, this is still controversial since previous evidence suggested that high-affinity TCR-like CAR, but not the low affinity one, demonstrated specific and potent cytotoxicity [42]. This is plausibly because of the fact that scFvs with high affinities to pMHC can decrease the threshold of antigen density required for T cell activation [43]. Petrausch and colleagues developed and used one of these high-affinity scFvs recognizing NY-ESO-1_157–165_/HLA-A*02:01. Intriguingly, the transduced T cells had a predominant effector memory phenotype, and showed an specific antitumor activity and cytokine secretion when stimulated with NY-ESO-1_157–165_ [44]. Overall, it seems that the researchers should ensure the optimum binding affinity of the antigen-recognition domain prior to designing TCR-like CAR T cells.

An expected advantage of TCR-like CARs or TCR-CARs is their capability in distinguishing neoepitopes from wild-type epitopes, as TCRs are able to specifically recognize tumor neoepitopes and spare wild-type peptides [45]. Thus, triggering tumor neoepitopes via effector cells expressing TCR-like CAR or TCR-CAR is increasingly garnering considerable attention. A close example is targeting of minor histocompatibility antigens (mHAgs), known as neoantigens’ therapeutic equivalents [46]. mHAgs are a group of immunogenic peptides that are originated from polymorphic genes and presented on the cell surface in association with class I or class II MHC molecules evoking strong alloreactivity [5]. Like neoepitopes, a subset of mHAgs can be strictly expressed on the hematopoietic malignant cells introducing them as tumor-specific antigens [46]. Inaguma et al., produced TCR-like CARs by introducing scFvs targeting the mHAg HA-1^H^ presented by HLA-A2. Similar to previous reports [40, 41], the authors showed that high-affinity scFvs (*K*_d_ = 19.9 nM) exerted lower cytotoxicity against target cells with low-density peptide/MHC complexes (~100 per cell) than the scFvs with moderate to low affinity (*K*_d_ = 446 nM) [47].

Another encouraging sign for the development of TCR-based CARs against neoepitopes is the feasibility of neoepitope-reactive TCR-T cells. In this regard, accumulating evidence suggests that TCR-Ts against neoepitopes such as those arising from K-Ras (NCT03190941) or TP53 mutations [48] have clinical benefit. Thus, it is anticipated that the benefits of neoepitope-reactive TCR-T cells will be extended to the TCR-like CAR/TCR-CAR T cells. Table 1 summarizes some examples of TCR-like CARs or TCR-CARs targeting the complex of epitope/neoepitope and HLA molecule.

**Table 1:** Some examples of the application of TCR-like CARs and TCR-CARs in human cancers.

Target antigen	Epitope type	HLA type	CAR ECD	CAR ICDs	Highlights of the study	Refs.
MELAN-A, TGFβR2	Wild-type, mutated	HLA-A2	TCR domains	CD28/CD3ζ	- Stable expression of TCR-CARs, even in CD3-free system such as the NK cells (for TGFβR2-specific TCR-CAR) - Efficient targeting and killing activity of TCR-CARs	[4]
NY-ESO-1, MAGE-A3	Wild-type, mutated	HLA-A*0201	Single variable domain of TCRβ chain	CD28/4-1BB/CD3ζ	- Stable expression of TCR-CARs on T cells - Selective binding of TCR-CAR to pMHC - Triggering T cells in the same manner as full TCRs	[36]
NY-ESO-1	Wild-type, mutated	HLA-A*0201	scFv	CD28/CD3ζ	- Despite soluble Fab specificity, CARs expressing the same scFv caused moderate lysis of targets independent of antigen - Lowering the affinity of the CAR for HLA-A2 restored the TCR-like CAR T cell’s epitope specificity and improved its cytolytic activity	[39]
WT1	Mutated	HLA-A*2402	scFv	CD3ζ/GITR	- TCR-like CAR T cells showed therapeutic efficacy - DCs loaded with the cognate antigen enhanced CAR T cells efficacy	[40]
WT1	Wild-type	HLA-A2	scFv	CD28/FcRγ	- In contrast to low-affinity αβ-TCRs, the high-affinity TCR-like CAR exhibited reduced activity and loss of specificity - Combination of high affinity and avidity of a TCR-like CAR displayed dramatic effects on the specificity	[41]
NY-ESO-1	Wild-type	HLA-A*0201	scFv	CD28/CD3ζ	- TCR-like CAR T cells had a predominant effector memory phenotype, and lysed target cells in an antigen-specific manner	[44]
HA-1^H^	Mutated	HLA-A*0201	scFv	CD28/CD3ζ	- High-affinity TCR-like CARs (*K*_d_ = 19.9 nM) exerted lower cytotoxicity in target cells with low-density peptide/MHC complexes (~100 per cell), when compared to the scFvs with moderate to low affinity (*K*_d_ = 446 nM) - CD8^+^ TCR-like CAR T cells were not necessarily CD8-dependent probably due to failure to form stable complexes with CD3	[47]
AFP	Mutated	HLA-A*0201	scFv	CD28/CD3ζ	- TCR-like CAR T cells selectively activated, released cytokines, and killed AFP^+^ target cells in vitro and in vivo.	[56]
WT1	Wild-type	HLA-A2	scFv	4-1BB/CD3ζ	- Affinity-enhanced scFv showed exquisite specificity towards WT1 epitope - Bivalent scFv-huIgG1-Fc fusion protein showed higher avidity to the epitope and induced cytolytic function of TCR-like CAR T/NK cells	[42]
PR1	Wild-type	HLA-A2	scFv	CD28/CD3ζ	- Efficient transduction and function of TCR-like CARs into PBMCs as well as T cells generated from umbilical cord blood (more permissive for HLA-mismatching) - TCR-like CAR T cells displayed preferential cytotoxicity against PR1^+^ leukemia cells	[57]

*ECD* extracellular domain, *ICD* intracellular domain.

## Limitations and concluding remarks

Multiple groups have found that patient-derived T cell repertoire recognizing neoepitopes are remarkably efficacious for personalized cancer immunotherapy [49, 50]. Following these encouraging outcomes, a concerted effort is currently in progress to develop endogenous TCR alternatives such as TCR-like CARs or TCR-CARs to extend the TCR specificity into the CAR platform while garnering the advantages of CAR platforms [4]. To this end, current studies have mainly focused on the improvement of methodologies used for neoepitopes identification in order to precisely identify tumor-specific neoepitopes. However, some limitations might hinder the success of neoepitope-directed TCR-like CAR or TCR-CAR therapies. First, the feasibility of this approach might be restricted to the tumors which tend to have a high mutational burden such as melanoma and lung cancer, but not common epithelial tumors that have much lower mutational loads [51]. Second, during tumor recurrence or relapse, it is plausible to observe a different profile and rate of neoepitopes expression [52]. Third, neoepitopes with the highest expression level and putative binding affinity to MHC molecules are not always suitable candidates, because they might not sufficiently exert neoepitope-reactive T cell response [53]. Therefore, in parallel with neoepitopes characterization in tumor cells, there is a need to validate neoepitope-reactive TCRs. As fourth limitation, TCR-CARs, especially those with higher binding affinity to pMHC, are potent in the redundant recognition of alternative targets and may cause subsequent toxicities owing to the “off-target” cross-reactivity [54]. Fifth, the majority of neoepitopes differ from one patient to another that limits their use to solely personalized therapies. However, this is being addressed by the identification of “shared neoepitopes” observed in a large subset of patients [55]. And finally, immune pressure by TCR-like CAR or TCR-CAR T cells can drive tumor cells to activate escape pathways and modulate the expression of target antigens [3]. Consequently, the ubiquitously tumor-expressed mutations and generation of novel predominant neoepitopes following the TCR-based CAR T therapies might further enhance the complexity of this treatment modality.

Collectively, while neoepitope targeting with engineered T cells carrying TCR-based chimeric receptors is an appealing strategy, candidate neoepitope or neoepitope-targeting domains should be selected with considerable caution. Moreover, the safety and tolerability of these modified cells are still speculative and further improvements are still needed to drive impressive clinical outcomes.
